# Impacts of RNA Mobility Signals on Virus Induced Somatic and Germline Gene Editing

**DOI:** 10.3389/fgeed.2022.925088

**Published:** 2022-06-09

**Authors:** Bliss M. Beernink, Ryan R. Lappe, Melissa Bredow, Steven A. Whitham

**Affiliations:** Department of Plant Pathology and Microbiology, Iowa State University, Ames, IA, United States

**Keywords:** CRISPR/Cas9, virus-induced gene editing, foxtail mosaic virus, potato virus X, barley stripe mosaic virus, tobacco rattle virus, *Nicotiana benthamiana*, *Zea mays*

## Abstract

Viral vectors are being engineered to deliver CRISPR/Cas9 components systemically in plants to induce somatic or heritable site-specific mutations. It is hypothesized that RNA mobility signals facilitate entry of viruses or single guide RNAs (sgRNAs) into the shoot apical meristem where germline mutations can occur. Our objective was to understand the impact of RNA mobility signals on virus-induced somatic and germline gene editing in *Nicotiana benthamiana* and *Zea mays*. Previously, we showed that foxtail mosaic virus (FoMV) expressing sgRNA induced somatic mutations in *N. benthamiana* and *Z. mays* expressing Cas9. Here, we fused RNA mobility signals to sgRNAs targeting the genes encoding either *N. benthamiana* phytoene desaturase (PDS) or *Z. mays* high affinity potassium transporter 1 (HKT1). Addition of *Arabidopsis thaliana* Flowering Locus T (AtFT) and *A. thaliana* tRNA-Isoleucine (AttRNA^Ile^) did not improve FoMV-induced somatic editing, and neither were sufficient to facilitate germline mutations in *N. benthamiana*. Maize FT homologs, Centroradialus 16 (ZCN16) and ZCN19, as well as AttRNA^Ile^ were found to aid somatic editing in maize but did not enable sgRNAs delivered by FoMV to induce germline mutations. Additional viral guide RNA delivery systems were assessed for somatic and germline mutations in *N. benthamiana* with the intention of gaining a better understanding of the specificity of mobile signal-facilitated germline editing. Potato virus X (PVX), barley stripe mosaic virus (BSMV), and tobacco rattle virus (TRV) were included in this comparative study, and all three of these viruses delivering sgRNA were able to induce somatic and germline mutations. Unexpectedly, PVX, a potexvirus closely related to FoMV, expressing sgRNA alone induced biallelic edited progeny, indicating that mobility signals are dispensable in virus-induced germline editing. These results show that PVX, BSMV, and TRV expressing sgRNA all have an innate ability to induce mutations in the germline. Our results indicate that mobility signals alone may not be sufficient to enable virus-based delivery of sgRNAs using the viruses, FoMV, PVX, BSMV, and TRV into cell types that result in germline mutations.

## Introduction

Targeted gene modification in plants has been widely used to study gene function and facilitate crop improvement. The CRISPR/Cas9 (clustered regularly interspaced short palindromic repeats (CRISPR)-associated protein 9) system is the most widely studied and utilized technology platform to facilitate plant genome engineering (El-Mounadi et al., 2020; Zhu et al., 2020; Dong et al., 2021; Wang et al., 2021; Zhang et al., 2021). CRISPR/Cas9 systems are adapted from naturally occurring bacterial immune systems. In genome engineering applications, sequence-specific single guide RNAs (sgRNA) are designed against the target sequence, and they form complexes with the Cas9 enzyme, which introduces an endonuclease-mediated double stranded break in the complementary DNA sequence (Jinek et al., 2012). The non-homologous end joining DNA repair pathway repairs the DNA breaks and typically introduces small insertions or deletions (indels) at the cleavage site. Non-homologous end joining-mediated repair is commonly used to generate loss-of-function alleles, while homology-directed recombination can be used to create precise DNA insertions, deletions, or base substitutions when a defined DNA repair template is provided (Xue and Greene, 2021). A major limitation of all genome-editing technologies, including CRISPR/Cas9, is that they are restricted to plants that can be transformed and regenerated (Mao et al., 2019; Venezia and Creasey Krainer, 2021). To expand the application of genome-editing systems toward plants that are recalcitrant to transformation, or expedite it in existing systems, new technologies are required to overcome the bottlenecks associated with conventional methods to deliver genome-editing components *in* *planta*.

Plant viruses have been extensively used to transiently overexpress or silence genes in model and crop plants. RNA and DNA viruses have also been modified to deliver sgRNAs systemically in transgenic plants expressing Cas9 resulting in somatic gene edits in plant tissues (Ron et al., 2014; Ali et al., 2015; Cai et al., 2015; Li et al., 2015; Yin et al., 2015; Cody et al., 2017; Ali et al., 2018; Gao et al., 2019; Hu et al., 2019; Mei et al., 2019). Virus-induced germline gene-editing systems have been demonstrated using the naturally seed transmissible viruses, tobacco rattle virus (TRV) (Ali et al., 2015; Ellison et al., 2020) and barley stripe mosaic virus (BSMV) (Li et al., 2021), in Cas9-expressing *N. benthamiana* and *Triticum aestivum*, respectively. Virus-delivered sgRNAs were capable of inducing edits in germline cells resulting in progeny possessing monoallelic and biallelic mutations at target sites (Li et al., 2021). RNA mobility elements, such as a truncated form of the *Arabidopsis thaliana* Flowering Locus T (AtFT) mRNA, were shown to promote the movement of mRNAs and viral RNAs into the meristem (Li et al., 2009), and fusion of *A. thaliana* tRNA-Isoleucine (AttRNA^Ile^) (Zhang and Ferré-D’Amaré, 2016) to sgRNAs has been shown to enhance cell-to-cell movement and traffic sgRNAs from somatic tissues to the shoot apical meristem (SAM). Notably, fusion of AtFT to sgRNAs in TRV increased the frequencies of both somatic and germline mutations in *N. benthamiana* (Ellison et al., 2020). In contrast, the fusion of TaFT, an FT homolog from *T. aestivum*, to sgRNA delivered by BSMV significantly hindered the number of edited progeny produced compared to the sgRNA alone (Li et al., 2021). Potato virus X (PVX) is a non-seed transmissible virus that is normally excluded from the SAM (Schwach et al., 2005). However, it has been shown that PVX expressing AtFT sequences fused to a GFP coding sequence was able to gain access to the SAM (Li et al., 2011). Accordingly, PVX carrying AtFT fused to the 3′ end of sgRNA sequences induced germline mutations in Cas9 *N. benthamiana* plants (Uranga et al., 2021).

Identifying and developing viral vectors capable of infecting a broad range of hosts is expected to expand the scope of accessible gene editing for economically important plants. Viruses belonging to the *Potexvirus* genus have been developed as viral vectors because of their small but modifiable genomes and large host ranges, including a number of monocotyledonous species (Lu et al., 2007; Liou et al., 2014; Minato et al., 2014; Mei et al., 2016). The potexvirus, foxtail mosaic virus (FoMV) is a positive-sense single stranded RNA virus that was reported to infect 35 dicotyledonous plants and 56 monocotyledonous plants, including economically important agricultural crop species such as *Z. mays*, *Sorghum bicolor*, and *T. aestivum* (Paulsen, 1977). FoMV was engineered for virus-induced gene silencing (VIGS) (Liu et al., 2016; Mei et al., 2016) and virus-mediated overexpression of proteins (Bouton et al., 2018; Mei et al., 2019), and it has also been demonstrated to deliver functional sgRNAs in *Z. mays*, *Setaria viridis*, and *N. benthamiana* (Mei et al., 2019; Zhang et al., 2020). However, the ability of RNA mobility sequences, such as FTs and tRNAs, to promote FoMV-induced somatic and germline mutations remains to be explored.

In this study, we fused AtFT and AttRNA^Ile^ to the 3′ end of sgRNA in FoMV and assessed somatic and germline genome edits in the *N. benthamiana* phytoene desaturase (*NbPDS*) gene. Additionally, we assessed the ability of FoMV-delivered sgRNAs to induce somatic and germline mutations in the *Z. mays* high affinity potassium transporter 1 (*ZmHKT1*) gene when fused to AtFT, AttRNA^Ile^, or maize FT homologs *Z. mays* centroradialis 8 (ZCN8), ZCN16, or ZCN19 (Danilevskaya et al., 2008). Fusion of sgNbPDS to AtFT or AttRNA^Ile^ did not affect the observed frequency of somatic genome edits compared to sgNbPDS alone, when delivered by FoMV in *N. benthamiana*. Fusions of sgZmHKT to ZCN16, ZCN19, or AttRNA^Ile^ resulted in notable improvements in the number of plants for which somatic genome edits were detected, but high variability precluded these from being significant when delivered by FoMV in *Z. mays*. Regardless of the sgRNA or mobility signal, all combinations utilized were insufficient to induce detectable germline mutations in progeny of either plant species. In a comparative approach to better understand the contribution of AtFT to heritable virus-induced gene editing in *N. benthamiana*, we found that TRV, PVX, and BSMV carrying sgRNA alone were all able to induce germline mutations under our conditions, unlike FoMV. Germline mutations were enhanced for TRV (as previously reported) when the sgRNA was fused to AtFT, contrasting with PVX and BSMV that produced gene edited progeny at similar frequencies independent of AtFT. Cumulatively, our results indicate that RNA mobility signals, such as FT, fused to sgRNAs are not sufficient to facilitate virus-induced germline mutations.

## Methods and Materials

### Plant Growth and Maintenance and Viral Inoculation

Transgenic *N. benthamiana* stably expressing Cas9 from *Streptococcus pyogenes* (SpCas9) (Baltes et al., 2015) were germinated on LC1 Grower’s Mix (Sungro) in a growth room maintained at 24°C with a 16 h photoperiod, and fertilized weekly with Peter’s Excel 15-5-15 (ICL Performance Products) at 300 parts per million (ppm). Four-week-old *N. benthamiana* plants were infiltrated with *Agrobacterium tumefaciens* strain GV3101 containing viral vectors suspended in an infiltration buffer containing 10 mM MgCl_2_, 10 mM MES, and 200 mM acetosyringone (pH 5.6) (Mei et al., 2019). Plants were grown to maturity at 22°C–25°C, with 16 h of light (320–350 μmol m^2^/s) and 47%–55% relative humidity. *Z. mays* seedlings stably expressing *Oryza sativa* codon optimized Cas9 (OsCas9) transgene (Jiang et al., 2013; Char et al., 2017) were germinated in LC1 Grower’s Mix (Sungro) and maintained at 22°C–23.5°C with a 16 h photoperiod (relative humidity between 47% and 55%), and fertilized weekly with Peter’s Cal-Mag Special (ICL Performance Products) 15-5-15 at 300 ppm. At 6 days post germination, *Z. mays* seedlings were injected with *A. tumefacians* strain GV3101 containing FoMV constructs suspended in 10 mM MgSO_4_ and 200 mM acetosyringone as described previously (Beernink et al., 2021). Plants were self-pollinated to obtain progeny.

### Viral Vector Construction

**FIGURE 1:**
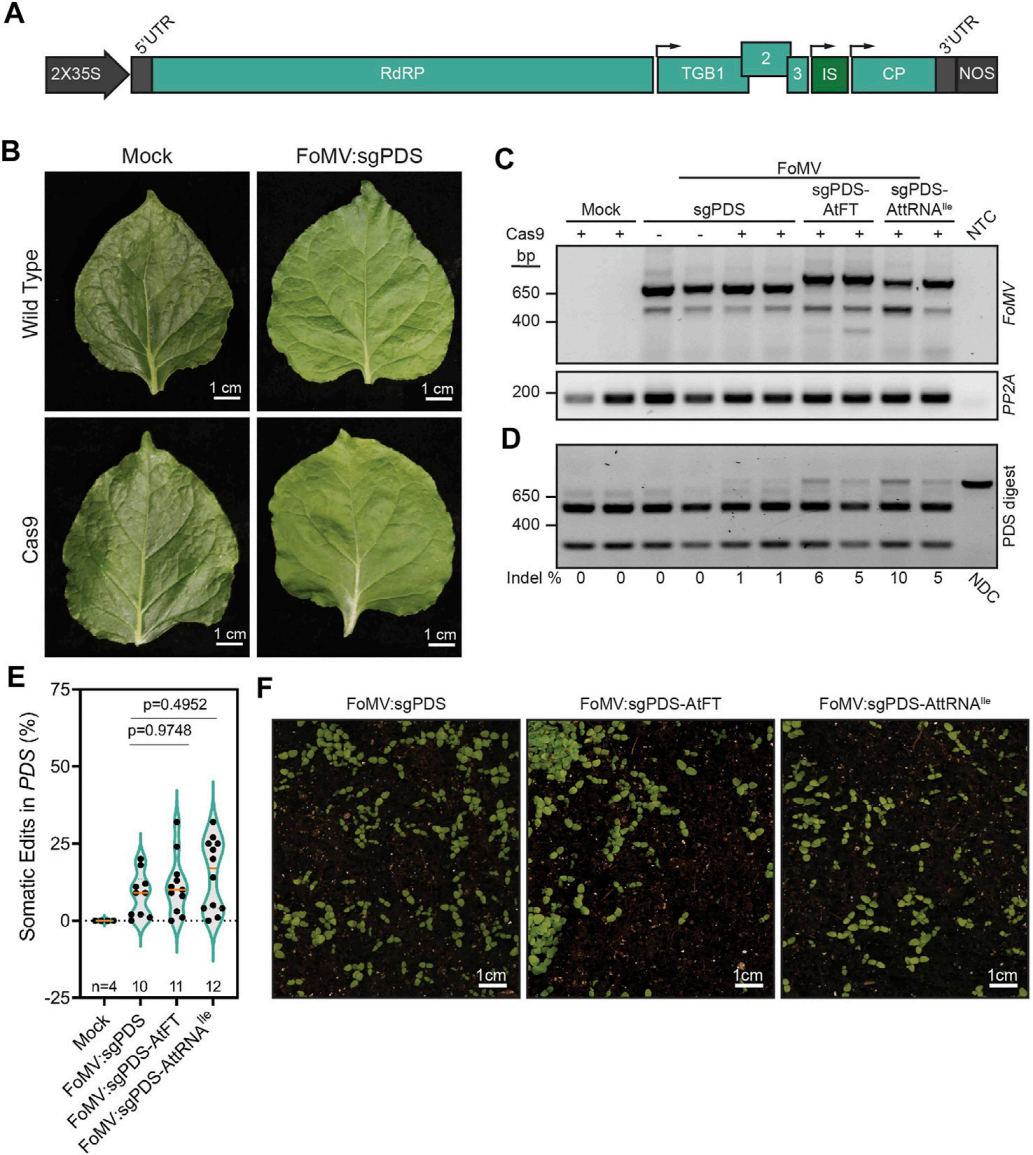
Effects of RNA mobility signals on FoMV-induced gene editing of *NbPDS* in *Nicotiana benthamiana*. **(A)** Schematic representation of the foxtail mosaic virus (FoMV) vector. The FoMV genome is transcribed under the control of a 2X cauliflower mosaic virus 35S promoter (35S) and the nopaline synthase (NOS) terminator. The FoMV genome consists of an RNA-dependent RNA polymerase (RdRP), triple gene block proteins (TGB1, 2, 3), and coat protein (CP), flanked by 5′ and 3′ untranslated regions (UTRs). sgRNAs or sgRNAs fused to RNA mobility signals are located in the insertion site (IS) between TGB3 and CP. Small black arrows above the FoMV genome represent the positions of the subgenomic promoters. **(B)** Images of systemic leaves of wild type or Cas9 *N. benthamiana* plants that were either mock-inoculated or inoculated with FoMV:sgNbPDS (PDS) 5 weeks post-inoculation. **(C)** RT-PCR amplification of FoMV in systemic leaf 8, approximately 5 weeks after inoculation. Primers flanking the IS of FoMV were used to demonstrate the stability of sgRNAs and sgRNAs fused to AtFT or AttRNA^Ile^. Intact FoMV:sgRNA is represented by a 571 bp amplicon, FoMV:sgRNA-AtFT is represented by a 673 bp amplicon, and FoMV:sgRNA-AttRNAIle is represented by a 646 bp amplicon. A 475 bp amplicon represents FoMV in which the sgRNA and mobility signal insert is fully deleted, amplicons between 475 bp and the applicable intact amplicon band size represent partial deletions of the insert. RT-PCR amplification of *N. benthamiana* *PP2A (PP2A)* serves as an internal control, and NTC is the no template control. Mock-inoculated Cas9 *N. benthamiana* serve as a negative control for FoMV infection, and FoMV:sgPDS infected wild type *N. benthamiana* serve as a negative control for somatic mutations. **(D)** A representative gel showing FoMV-induced somatic mutations (see Supplementary Figure S2 for additional gels). A fragment of the *NbPDS* gene containing the sgNbPDS target site was PCR amplified from genomic DNA isolated from systemic leaf tissue of wild type or Cas9-expressing transgenic *N. benthamiana* infected with FoMV containing sgNbPDS or sgNbPDS fused to AtFT or AttRNA^Ile^. The resulting amplicons were digested with *Nco*I restriction endonuclease to test for the presence of indels disrupting the restriction site. The non-digested control (NDC) represents the wild type 797 bp *NbPDS* amplicon and undigested amplicons containing indels. The absence of indels at the *Nco*I site results in fragments resolving at 256 and 541 bp. The percentage of somatic mutations detectable for each represented plant is noted underneath the gel image, ratios were calculated from digested and non-digested fragment intensities obtained using ImageJ. Mock-inoculated transgenic Cas9 *N. benthamiana* serve as a negative control for FoMV infection, and FoMV:sgPDS infected wild type *N. benthamiana* served as a negative control for somatic mutations. **(E)** A summary of all *NbPDS* amplicons containing indels induced by FoMV:sgNbPDS, FoMV:sgNbPDS-AtFT, and FoMV:sgNbPDS-AttRNAIle are reported as percentages in a violin plot, ratios are calculated from fragment intensities using ImageJ, data points are reported from two replicates with n representing the number of plants. The median and first and fourth quartiles for each data set are represented by a solid orange line and dotted green lines, respectively. Pairwise *p*-values are indicated from statistical analysis by one-way ANOVA and subsequent post hoc Student’s t-test for each comparison. **(F)** Representative images of progeny from parental *N. benthamiana* plants infected with FoMV containing sgNbPDS, sgNbPDS-AtFT, or sgNbPDS-AttRNAIle. Two independent replicates of this experiment were conducted.

FoMV has a positive-sense single stranded RNA genome that is 6.2 kilobases (kb) in length, encoding five different proteins from five open reading frames (ORFs) (Bancroft et al., 1991) (Figure 1A). ORF1 encodes the RNA-dependent RNA polymerase (RdRP) (Rouleau et al., 1993) that is essential for genome replication and the transcription of two sub-genomic mRNAs. ORFs 2, 3, and 4 are collectively known as the triple gene block (TGB) movement proteins (Samuels et al., 2007; Bruun-Rasmussen et al., 2008), and ORF5 encodes the coat protein (CP), which also aids in cell-to-cell movement (Lough et al., 2000). In order to generate vectors capable of delivering sgRNAs, a modified version of FoMV was constructed containing a Cas9 scaffold sequence (Cas9scaf) (Jinek et al., 2012) inserted at MCSI located between ORF 4 and 5, as described previously (Mei et al., 2019). An *Mlu*I restriction site was added upstream of the Cas9scaf sequence (FoMV-MluI-Cas9scaf) for seamless cloning of CRISPR RNAs (crRNAs) to make sgRNA constructs, as described below. Sequences from RNA mobility signals, consisting of the 102 bp truncated AtFT sequence (Li et al., 2009, Li et al., 2011) (Supplementary Table S1) were cloned at the 3′ end of sgRNA sequences as this orientation has been shown to successfully induce both somatic and germline mutations using TRV and BSMV (Ellison et al., 2020; Li et al., 2021). The 102 bp fragments of ZCN8, ZCN16, and ZCN19 were selected based on alignment with the mRNA sequence of AtFT. Synthetic double stranded DNA fragments (gBlocks) (Integrated DNA Technologies, Coralville, Iowa, United States) for MluI-Cas9scaf, MluI-Cas9scaf-AtFT, MluI-Cas9scaf-AttRNAIle and MluI-Cas9scaf-ZCN8/19/16 (Supplementary Table S2) were cloned into MCSI of FoMV, digested with *Bsu*36I and *Psp*OMI and subsequently dephosphorylated, using NEBuilder HiFi DNA assembly (New England Biolabs, Ipswich, MA, United States). *NbPDS* (Niben101Scf01283g02002.1 and Niben101Scf14708g00023.1) and *ZmHKT1* (GRMZM2G047616) genes were selected as targets for CRISPR-Cas9-mediated gene editing in *N. benthamiana* and *Z. mays*, respectively, as described previously (Mei et al., 2019). crRNAs for *NbPDS* and *ZmHKT1* (Supplementary Table S2) were generated as synthetic DNA oligonucleotides (Integrated DNA Technologies, Coralville, IA, United States) and cloned into FoMV-MluI-Cas9Scaf at the *Mlu*I restriction site in the sense orientation (Figure 1A). Briefly, FoMV-MluI-Cas9Scaf vectors were digested with *Mlu*I, dephosphorylated, and sgNbPDS and sgZmHKT oligonucleotides were incorporated by NEBuilder HiFi DNA assembly (New England Biolabs, Ipswich, MA, United States) to generate FoMV:sgNbPDS and FoMV:sgHKT with 3′ fusion of AtFT, AttRNA^Ile^, ZCN8, ZCN16 and ZCN19 mobility signals (Supplementary Table S1).

TRV:sgNbPDS and TRV:sgNbPDS-AtFT have been previously described (Ellison et al., 2020). pYL254:PVX viral constructs (Liu et al., 2002) were modified to contain a sgRNA targeting *NbPDS* and the 102 bp RNA mobility signal from AtFT. sgRNA DNA sequences, with or without AtFT, were amplified from FoMV constructs with primers sgPDS-F/AtFT102-R and sgPDS-F/Cas9Scaf-R, respectively. The amplicons were cloned into the Gateway compatible entry vector, pCR8/GW/TOPO, using a TA Cloning Kit (ThermoFisher Scientific, Waltham, MA, United States). Subsequent recombination into the PVX destination vector, pYL254, was performed using Gateway LR clonase II Enzyme Mix (Thermo Fisher Scientific, Waltham, MA, United States) in order to generate PVX:sgNbPDS and PVX:sgNbPDS-AtFT (Supplementary Figure S1). All vectors were confirmed by Sanger sequencing (Iowa State University DNA Core Facility) (Supplementary Figure S1). BSMV constructs for gene editing in *N. benthamiana* were generated by integrating a sgRNA targeting *NbPDS* downstream of the BSMVɣb ORF (Yuan et al., 2011) using NEBuilder HiFi DNA assembly (New England Biolabs, Ipswich, MA, United States) as described above. The BSMVɣb:sgNbPDS construct was further modified to incorporate the truncated RNA mobility signal from AtFT on the 3′ end of the sgRNA to produce BSMVɣb:sgNbPDS-AtFT. BSMVɣb:sgNbPDS or BSMVɣb:sgNbPDS-AtFT were co-expressed with BSMVα and BSMVβ in order to launch the BSMV-sgRNA system (Supplementary Figure S1). Oligonucleotides and synthetic double stranded DNA fragments (gBlocks) (IDT, Coralville, IA, United States) used for vector construction are listed in Supplementary Table S2.

### Reverse Transcription-PCR Analysis

Reverse transcription (RT)-PCR was conducted to evaluate systemic viral infection and stability of sgRNA and mobility sequences. Leaf samples were harvested from *N. benthamiana* and *Z. mays* plants 5–6 weeks after inoculation as indicated in figure legends. RNA was extracted using Trizol reagent (Thermo Fisher Scientific, Waltham, MA, United States), and 2 μg of total RNA was added to the first-strand cDNA synthesis using the Maxima H Minus cDNA Synthesis Kit (Thermo Fisher Scientific, Waltham, MA, United States). FoMV was detected by RT-PCR using 5AmuS2 and 5AmuA2 primers (Supplementary Table S2) designed to span the insertion site in MCS1 (Mei et al., 2019). Primers pYL254-F and pYL254-R were used for the detection of PVX (Supplementary Table S2). Primers TRV2-F and TRV2-R were used for the detection of TRV RNA 2. Primers BSMVɣb-F and BSMVɣb-R were used for the detection of BSMVɣ. Primers NbPP2A-F and NbPP2A-R (Ellison et al., 2020) were used as internal reference controls for *N. benthamiana*, and primers ZmActin-F and ZmActin-R were used for internal controls for *Z. mays* (Supplementary Table S2).

### Detection of Mutations in *N. benthamiana* and *Z. mays* Target Genes

SgRNAs for *NbPDS* and *ZmHKT1* gene editing were designed to target sequences containing restriction enzyme recognition sites 3-4 bps upstream of protospacer adjacent motifs in order to detect gene edits by enzyme digestion of amplicons as described previously (Xing et al., 2014; Mei et al., 2019). Systemic leaves were harvested from *N. benthamiana* plants approximately 5 weeks post-inoculation (leaf 8). For *Z. mays*, leaf samples were collected at approximately 6 weeks post-inoculation from leaves showing symptoms of viral infection (leaf 7–9). Each leaf sample was divided at the time of collection for DNA and RNA extraction.

Genomic DNA was extracted from *N. benthamiana* and *Z. mays* leaf tissue using the cetyltrimethylammonium bromide (CTAB) method (Murray and Thompson, 1980). Amplicons spanning the sgRNA target sequences were generated using Platinum SuperFi II PCR Master Mix (Thermo Fisher Scientific, Waltham, MA, United States) using primers NbPDS-F and NbPDS-R for *NbPDS*, or ZmIDT-F0 and ZmIDT-R0 for *ZmHKT1* (Supplementary Table S2). *NbPDS* and *ZmHKT1* amplicons were digested with *Nco*I and *Xcm*I, respectively, and resolved on a 1% agarose gel. The pixel intensities of digested (unedited) and undigested (edited) DNA fragments were quantified using ImageJ software, and then the percentage of edits was calculated by dividing the non-edited fragment values by the sum of the non-edited and edited fragment values as described in Mei et al. (2019).

To determine if mutations were heritable, seeds were collected from the parent plants and screened for gene edits. Successful biallelic editing of *NbPDS* in *N. benthamiana* seedlings results in an albino phenotype which can be visually quantified on perlite-free LC1 Grower’s Mix (Sungro). The number of seedlings screened was estimated based on the seed weight (1,000 seed/78.4 mg). In maize, individual plants were self-pollinated in order to obtain progeny to screen for gene edits. Since *ZmHKT1* editing does not result in a visual phenotype, *Z. mays* seedlings were screened using restriction endonuclease digestion of genomic DNA PCR amplicons as described above.

## Results

### Foxtail Mosaic Virus-Induced Somatic Editing in *N. benthamiana* is Not Affected by RNA Mobility Signals

In order to evaluate the impact of mobile RNA elements on FoMV-driven sgRNA delivery, we assessed *NbPDS* gene editing in systemic *N. benthamiana* leaves at 5 weeks post-inoculation. FoMV carrying the sgRNA targeting *NbPDS* caused no obvious infection symptoms and did not induce photobleaching associated with PDS loss-of-function mutations (Kumagi et al., 1995) in SpCas9-expressing *N. benthamiana* leaves when compared to mock-inoculated plants (Figure 1B). The lack of photobleaching is consistent with our previous observations (Mei et al., 2019). However, RT-PCR analysis confirmed successful systemic viral infection in leaves of plants inoculated with FoMV:sgRNA and FoMV:sgRNA-RNA mobility signal constructs (Figure 1C). Amplification of the viral genome spanning the sgNbPDS-RNA mobility signal insertion site indicated that gene editing components, with or without mobility signals, were stably expressed at 5 weeks post-infection. While there is some deletion of the inserts, the majority of the viral population retains the inserted sequence (Figure 1C). Despite the apparent lack of photobleaching, somatic edits in *NbPDS* were detected in these same leaves infected with FoMV:sgNbPDS, corresponding to the loss of the *Nco*I endonuclease recognition site in the sgRNA target sequence and undigested PCR amplicons (Figure 1D). Quantification of digested and undigested PCR amplicons using ImageJ software indicated that FoMV:sgRNA induced low levels of mutations (Figure 1E), which is in line with the lack of observable photobleaching (Figure 1B). Fusion of the AtFT or AttRNA^Ile^ RNA mobility signals to the 3′ end of the sgRNA resulted in an average of 11% (*n* = 11) and 15% (*n* = 12) somatic gene editing respectively, compared with 8% for sgRNAs alone (*n* = 10) (Figure 1E; Supplementary Figure S2). However, these plants also did not display photobleaching in leaves in which somatic editing was occurring (Supplementary Figure S3A).

### RNA Mobility Signals Do Not Promote Induction of Germline Mutations in the Context of FoMV in *N. benthamiana*

To determine if FoMV is capable of inducing germline mutations in *N. benthamiana*, we collected seed from 4–6 plants per replicate, per permutation, of FoMV:sgNbPDS either absent for or fused to mobility signals exhibiting somatic gene edits and screened them for white seedlings, which would be indicative of biallelic gene edits in *NbPDS*. No albino seedlings (from a total of 14,800 progeny screened) were detected in two independent experiments (*n* = 10) (Figure 1F), indicating that FoMV:sgNbPDS was not capable of inducing mutations in germline cells of *N. benthamiana*. Fusion of mobile RNA elements to the 3′ end of sgRNAs delivered by TRV and the potexvirus PVX has been attributed to the movement of sgRNA transcripts to the SAM (Ellison et al., 2020; Li et al., 2021; Uranga et al., 2021) and biallelic mutations in *NbPDS* resulting in progeny exhibiting an albino phenotype. Thus, we screened between 1,000 and 1,800 progeny from each FoMV:sgNbPDS-AtFT (*n* = 11, 15,000 total seedlings) and FoMV:sgNbPDS-AttRNAIle (*n* = 12, 16,800 total seedlings) infected plant with somatic mutations for the albino phenotype, but none were observed (Figure 1F). To determine if FoMV induced monoallelic gene edits in *NbPDS*, the target sequences in progeny from six FoMV:sgNbPDS infected parent plants (*n* = 41), four FoMV:sgNbPDS-AtFT parent plants (*n* = 25), and four FoMV:sgNbPDS-AttRNAIle parent plants (*n* = 10) were sequenced, however neither monoallelic nor biallelic edits were observed (Supplementary Figure S4). Together, our data suggest that the fusion of AtFT or AttRNA^Ile^ to the 3′ end of sgNbPDS does not affect editing in *N. benthamiana*, nor does it enable this FoMV clone to overcome barriers to inducing mutations that can be passed through the germline.

### ZCNs and AttRNA^Ile^ Enhance Somatic Gene Editing in *Z. mays* but Do Not Promote Foxtail Mosaic Virus-Driven Gene Editing in Germline Cells

**FIGURE 2:**
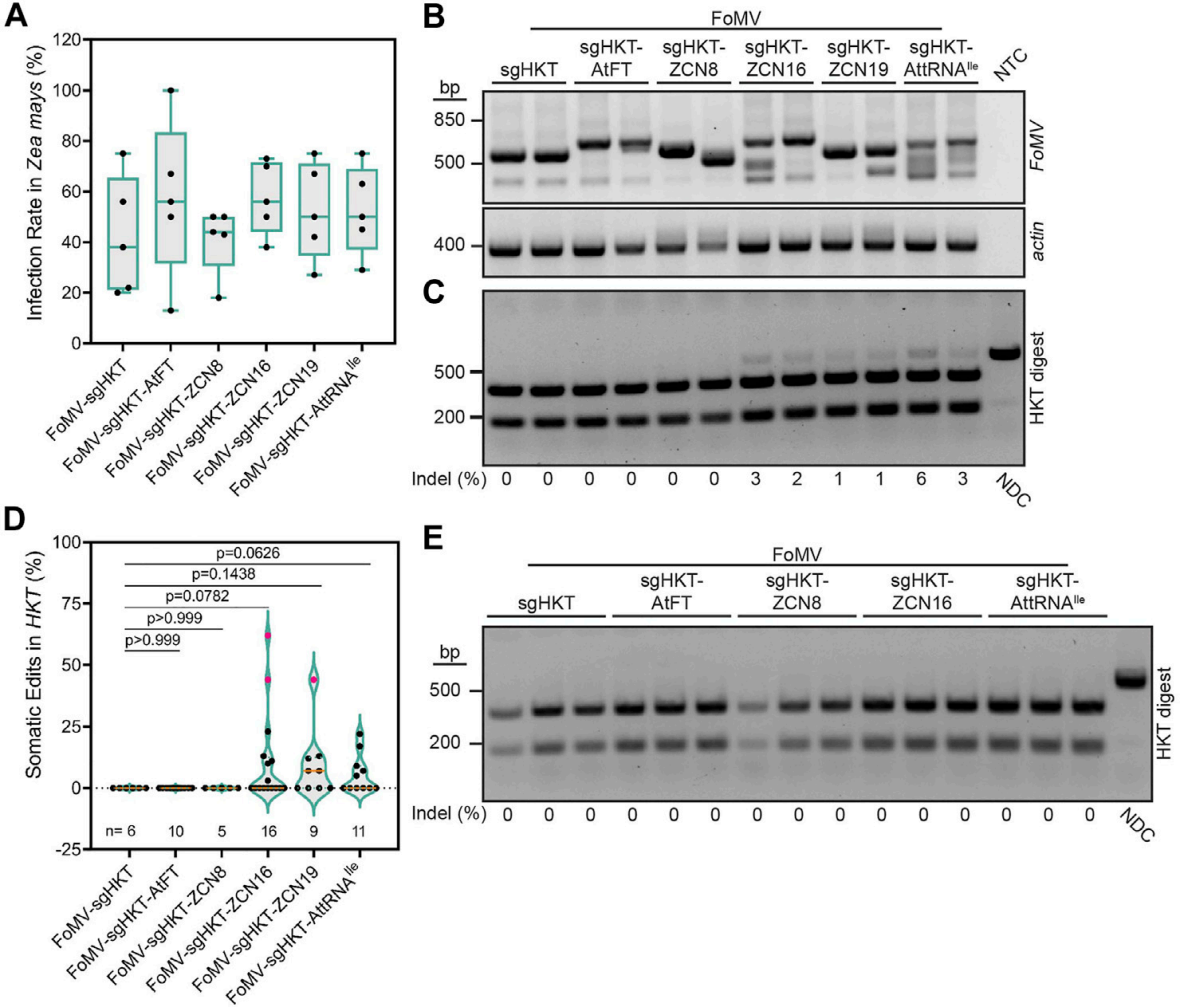
Effects of RNA mobility signals on FoMV-induced mutations in *ZmHKT1*. **(A)** Percentage of Cas9 maize plants displaying systemic FoMV infection 6 weeks post-injection, as determined by RT-PCR. Data are represented as box plots indicating the 25%–75% interquartile range, split by a median line. Whiskers represent maximum and minimum values. A one-way ANOVA indicated no significant differences in infection rates between treatments (*p* = 0.7024). **(B)** RT-PCR analysis of FoMV in systemic *Z. mays* leaves 6 weeks post-infection. Primers were designed to flank the insertion site to assess stability of sgRNA and sgRNA-mobility signal insertions. Intact FoMV:sgRNA is represented by a 571 bp amplicon, FoMV:sgRNA-AtFT, FoMV:sgRNA-ZCN8, FoMV:sgRNA-ZCN16, and FoMV:sgRNA-ZCN19 are represented by a 673 bp amplicon, and FoMV:sgRNA-AttRNAIle is represented by a 646 bp amplicon. ZmActin was used as an internal control, and NTC is the no template control. **(C)** Representative gel of FoMV-induced somatic mutations in *ZmHKT1* (see Supplementary Figure S2 for additional gels). A fragment of *ZmHKT1* containing the sgZmHKT target site was PCR amplified from genomic DNA isolated from systemic leaf tissue, 6 weeks post-inoculation. The resulting amplicons were digested with *Xcm*I restriction endonuclease to assess the presence of indels disrupting the restriction site. The percentage of somatic mutations detected for each plant is provided underneath the gel image, and were calculated from fragment intensities using ImageJ. Data points are from two replicates with n representing the number of plants. The non-digested control (NDC) represents the wild type 732 bp *ZmHKT1* amplicon and undigested amplicons containing indels. The absence of indels at the *Xcm*I site results in fragments resolving at 228 and 504 bp. **(D)** Percentage of somatic mutations in *ZmHKT1* amplicons digested with *Xcm*I were quantified by ImageJ to calculate the ratio of non-digested to digested amplicons. Results are reported as violin plots split by a median line, with dotted lines representing the first and fourth quartiles, data points are reported from two replicates. Pairwise *p*-values are indicated from statistical analysis by one-way ANOVA and post hoc Student’s t-test for each comparison. Pink data points were determined to be outliers using the ROUT method with Q = 1%. **(E)** Germline mutations in *Z. mays* progeny produced by parent plants infected with the indicated FoMV constructs. The NDC and mutant *ZmHKT1* amplicons migrate to 732 bp, whereas amplicons containing no mutations are fully digested by *Xcm*I producing fragments that resolve at 228 and 504 bp. Experiments were conducted two times.

Concurrent with gene editing experiments in *N. benthamiana*, we investigated if the previously established FoMV-enabled gene editing system (Mei et al., 2019) could be modified to generate germline mutations in *Z. mays*. Although AtFT was reported to enhance TRV-induced gene editing in *N. benthamiana*, TaFT resulted in a more striking photobleaching phenotype in *T. aestivum* leaves compared to AtFT when they were fused to sgRNA targeting *T. aestivum* *PDS* using BSMV (Li et al., 2021). FT homologs have also been identified in additional monocotyledonous crop plants including *O. sativa* (Hd3a) (Tamaki et al., 2007), *S. bicolor* (SbFT) (Wolabu et al., 2016), and *Z. mays* (ZCN) (Danilevskaya et al., 2008; Lazakis et al., 2011). However, the ability to promote germline editing using these FT homologs fused to sgRNA delivered by a virus has not been evaluated. For this reason, ZCN8, ZCN16, and ZCN19 (Danilevskaya et al., 2008), in addition to AtFT and AttRNA^Ile^, were used to assess the effect of mobile RNA elements on somatic and germline gene editing. No difference was observed in the ability of FoMV:sgZmHKT constructs to systemically infect OsCas9-expressing *Z. mays* (Figure 2A). Additionally, we confirmed the production of sgZmHKT via a unique subgenomic RNA in infected systemic *Z. mays* leaves by rapid amplification of cDNA ends (RACE) followed by Sanger sequencing (Supplementary Figure S5). RT-PCR analysis indicated that FoMV carrying sgRNA-RNA mobility signal fusions remained stable in systemic leaves at 6 weeks post-inoculation, with the exception of FoMV:sgZmHKT-ZCN8 which suffered insert deletions in all plants (Figure 2B; Supplementary Figure S6). Because *ZmHKT1* loss of function does not result in a visual phenotype (Mei et al., 2019), somatic gene editing in *ZmHKT1* was evaluated in systemic leaves displaying symptoms of viral infection. FoMV:sgZmHKT is capable of generating low levels of somatic edits in *Z. mays*, which are nearly undetectable by restriction digest (Mei et al., 2019) (Figure 2C). Although infection with FoMV:sgZmHKT-AtFT did not produce any detectable mutations, ZCN16, ZCN19, and AttRNA^Ile^ increased somatic gene editing to an average of 10.3%, 9.2%, and 5.5%, respectively (Figure 2D; Supplementary Figure S2). Fusion of ZCN8 to sgZmHKT did not aid somatic gene editing, presumably due to the low insert retention of FoMV constructs harboring this mobility signal (Supplementary Figure S6). We were able to generate self-crosses of FoMV:sgZmHKT, FoMV:sgZmHKT-ZCN8, FoMV:sgZmHKT-ZCN16 and FoMV:sgZmHKT-AttRNA^Ile^ plants in which FoMV infection was confirmed; seedlings were screened for mutations by PCR and digestion of the amplicons with *Xcm*I restriction endonuclease (*n* = 28, *n* = 57, *n* = 69, and *n* = 38, respectively), however, none of these progeny plants carried mutations in *ZmHKT1* (Figure 2E). Therefore, we conclude that while ZCN16 and AttRNA^Ile^ modestly enhance sgRNA delivery, they are not sufficient to promote access to meristematic tissues sufficient to induce germline mutations in *Z. mays*.

### Evaluating the Effect of Mobility Signals on Somatic and Germline Using Additional Viral Vector Delivery Systems

**FIGURE 3:**
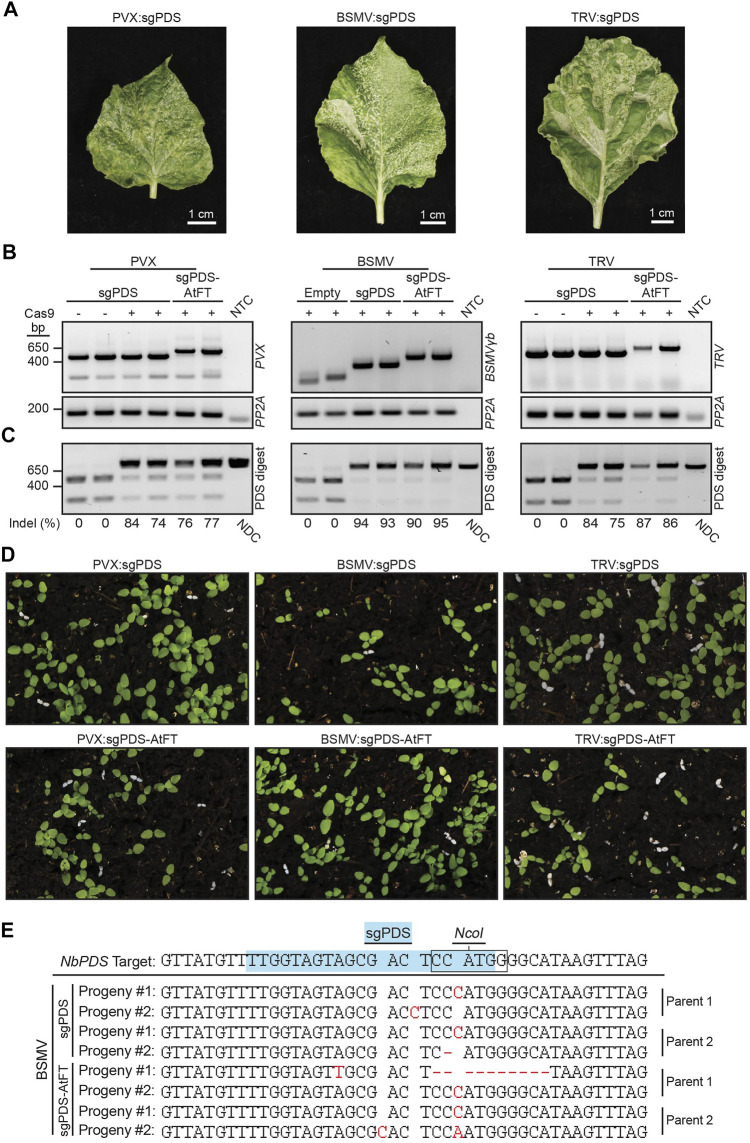
Somatic and germline mutations in *NbPDS* induced by BSMV, PVX or TRV containing a sgRNA alone or fused to truncated AtFT. **(A)** Systemic leaves of Cas9 *N. benthamiana* plants infected with BSMV:sgNbPDS, PVX:sgNbPDS, and TRV:sgNbPDS. **(B)** RT-PCR amplification of the indicated virus spanning sgRNA insertion sites from systemic parental leaf tissue. *NbPP2A* was used as an internal control. **(C)** *NbPDS* amplicons from systemic leaf DNA of plants infected with the indicated viral constructs following digestion with *Nco*I. Digestion of unedited *NbPDS* amplicons generates two fragments migrating to 256 and 541 bp. Non-digested fragments represent *NbPDS* editing due to disruption of the *Nco*I site, and have similar size to the non-digested control (NDC) amplicon (797 bp). For PVX and TRV, PVX:sgPDS and TRV:sgPDS infection of wild type *N. benthamiana* represents the negative control for somatic mutations. For BSMV experiments, the negative control for somatic mutations was Cas9 *N. benthamiana* infected with BSMV with no sgPDS. **(D)** Representative images of progeny from NbCas9 plants infected with BSMV:sgNbPDS, PVX:sgNbPDS, and TRV:sgNbPDS, with or without mobility signals. White seedlings represent progeny with biallelic mutations disrupting *NbPDS*. **(E)** Sanger sequencing results for white *N. benthamiana* seedlings produced from parent plants infected with BSMV:sgNbPDS or BSMV:sgNbPDS-AtFT. The blue highlighted sequence identifies the sgNbPDS crRNA target sequence, and the box indicates the *Nco*I restriction endonuclease recognition site. Red letters or dashes represent inherited indels observed as base additions or deletions in progeny, respectively. A single replicate with seven plants was performed with TRV, two independent replicates were performed with 21 plants total for BSMV, and three independent experiments were conducted with 23 plants total for PVX.

Our cumulative data suggested that the addition of RNA mobile elements such as FTs and AttRNA^Ile^ alone are not sufficient to permit FoMV-induced germline editing in *N. benthamiana* or *Z. mays*. We were curious if the addition of mobile RNA elements to sgRNAs were necessary for germline mutations in previously described systems producing biallelic gene edited progeny. Therefore, we conducted a series of comparative tests with additional viral delivery systems in Cas9 *N. benthamiana*. The TRV and PVX systems were shown to induce germline mutations in Cas9 *N. benthamiana* and BSMV in Cas9 *T. aestivum* (Ellison et al., 2020; Li et al., 2021; Uranga et al., 2021). PVX was particularly interesting to us, because it is a potexvirus like FoMV, and the AtFT mobility signal is reported to be functional in the context of PVX (Uranga et al., 2021). The ability of the PVX sgRNA delivery system to induce heritable biallelic mutations in *NbPDS* has not been previously tested. Accordingly, we generated PVX:sgNbPDS and PVX:sgNbPDS-AtFT clones and monitored somatic and germline mutations in *N. benthamiana*. PVX:sgNbPDS produced a photobleaching phenotype in systemic leaves (Figure 3A) associated with high levels of somatic mutations in *NbPDS* (67%, *n* = 9) (Figures 3B,C; Supplementary Figure S2). Similarly, PVX:sgRNA-AtFT generated an average of 58% (*n* = 12) somatic mutations of *NbPDS* and a moderate degree of photobleaching (Figures 3A–C; Supplementary Figure S2). Of the progeny from plants infected with PVX:sgNbPDS and PVX:sgNbPDS-AtFT, 0.00%–4.44% (*n* = 16,200) and 0.0%–2.83% (*n* = 21,600), respectively, contained biallelic mutations identified visually as albino seedlings (Figures 3B,D). Amplicon sequencing of these seedlings confirmed germline mutations in *NbPDS*, predominantly as small indels consisting of single nucleotide substitutions, insertions, or 1–12 base pair deletions (*n* = 20) (Supplementary Figure S4). These observations indicate that the addition of AtFT is not essential or beneficial (three independent experiments, two-tailed *t*-test, *p* = 0.2716; *p* = 0.5057; *p* = 0.6466) for germline editing in this system.

We also compared somatic and germline mutation efficiency of BSMV:sgNbPDS with BSMV:sgNbPDS-AtFT, which were previously reported not to induce germline mutations in Cas9 *N. benthamiana* (Li et al., 2021). BSMV:sgNbPDS and BSMV:sgNbPDS-AtFT induced an average of 82% and 90% (*n* = 11, *n* = 12) somatic mutations in *NbPDS* in the systemic leaves, respectively, with leaves displaying a high level of photobleaching including white leaf sectors (Figures 3A–C; Supplementary Figure S2). Germline mutations were evaluated first by determining the percentage of albino progeny produced by each plant. BSMV:sgNbPDS-infected plants produced 0.0%–2.22% (*n* = 19,800) progeny with biallelic mutations in *NbPDS* (Figure 3D), and the addition of AtFT to BSMV:sgNbPDS produced a similar result of 0.0%–1.94% (*n* = 21,600) (Figure 3D) (two independent experiments, two-tailed *t*-test, *p* = 0.4580; *p* = 0.3561). To corroborate these mutations, PCR amplicon products from albino seedling genomic DNA samples were sequenced. Small indels including single nucleotide substitutions or insertions, or 1–10 nucleotide deletions were identified 3-4 nucleotides upstream of the protospacer adjacent motif in nine progeny obtained from three parent plants infected with BSMV:sgPDS, and eight progeny obtained from three parent plants infected with BSMV:sgPDS-AtFT from two independent experiments (Figure 3E; Supplementary Figure S4). Collectively this data indicates that under our experimental conditions BSMV can successfully induce germline mutations in Cas9 *N. benthamiana*, seemingly without notable contribution from AtFT.

Additionally, we included TRV for comparative analysis in the evaluation of the impact of AtFT on viral vector sgRNA delivery *in planta*. *N. benthamiana* plants infected with either TRV:sgNbPDS or TRV:sgNbPDS-AtFT produced a strong photobleaching phenotype resulting from high levels of somatic mutations in systemic leaves (73%, *n* = 4; and 91%, *n* = 7, respectively) (Figures 3A–C; Supplementary Figure S2). This is relatively consistent with the reported data for TRV, where TRV:sgRNA induced an average of 61% somatic edits, and TRV:sgNbPDS-AtFT induced an average of  90% somatic edits (Ellison et al., 2020). An average of 5.5% (*n* = 4,400) of progeny produced by TRV:sgNbPDS-infected plants contained biallelic mutations in *NbPDS* compared to a published average of 19% of progeny containing either monoallelic or biallelic germline mutations with this same construct (Ellison et al., 2020). The addition of AtFT to TRV:sgNbPDS increased the number of progeny displaying biallelic gene edits to 15.4% (*n* = 6,000) (Figure 3), small indels consisting mainly of single nucleotide substitutions, insertions or deletions were confirmed by Sanger sequencing (*n* = 19) (Supplementary Figure S4). Our data indicate that TRV-based delivery of sgNbPDS is robust and enables high levels of somatic and germline mutations in Cas9 *N. benthamiana*, demonstrating a consistent pattern with published data. Based on these results, we conclude that the conditions of our experiments were conducive for heritable virus-induced gene editing and that the inability of our FoMV:sgRNA delivery system to produce biallelic edited progeny is not the product of unsuitable experimental conditions.

## Discussion

The use of RNA mobility sequences, such as AtFT and AttRNA^Ile^, were previously shown in the context of TRV and PVX to augment or enable delivery of sgRNAs into cell types that give rise to progeny with germline mutations. Here, we were interested in the possibility of extending these findings to FoMV with the goal of heritable virus-induced gene editing in maize. We took a comparative approach to assess the effectiveness of the RNA mobility signals in FoMV versus the TRV, PVX, and BSMV systems in *N. benthamiana*. We found that the RNA mobility signals tested did not enhance the frequency of somatic gene editing induced by FoMV (Figure 3) as previously observed for TRV and PVX (Ellison et al., 2020; Uranga et al., 2021), nor did the addition of these signals to sgRNAs enable it to induce biallelic mutations in the germline (Figure 1). Fusion of AttRNA^Ile^, ZCN16 and ZCN19 to sgRNA increased average somatic editing efficiency in *Z. mays*, but these constructs did not induce heritable monoallelic or biallelic mutations in *ZmHKT1*. The choice of mobility signals was important for improving somatic editing efficiency with AtFT affording no benefit to FoMV-sgRNA in *Z. mays* (Figure 2). These data show that neither FoMV:sgRNA nor FoMV:sgRNA bearing mobility signals were sufficient to induce gene editing in germline tissues at a detectable frequency in *N. benthamiana* or *Z. mays*. This is in contrast to PVX, BSMV, and TRV, which under our conditions, were all capable of delivering sgRNA such that somatic and germline cells were edited both with and without fusions to RNA mobility signals in *N. benthamiana*. Moreover, TRV:sgRNA was the most efficient at inducing heritable biallelic mutations in *NbPDS* and was most impacted by the addition of AtFT.

Some viral sgRNA delivery systems appear to have the innate capacity to induce germline mutations. For example, TRV was previously reported to induce germline mutations when delivering a sgRNA alone in *N. benthamiana* (Ali et al., 2015). BSMV expressing sgRNA alone was also shown to efficiently induce germline mutations in *T. aestivum*, but not in *N. benthamiana* (Li et al., 2021). Similar to BSMV, a previous report indicated that PVX expressing sgRNA alone was insufficient to induce germline mutations in *N. benthamiana* (Uranga et al., 2021). We found that BSMV and PVX, like TRV, are capable of inducing biallelic mutations transmitted to progeny in *N. benthamiana* without the use of RNA mobility signals under our conditions (Figure 3). The results with these three viruses are in stark contrast to FoMV, which did not induce biallelic mutations in the germline of *N. benthamiana* or *Z. mays*, even with the fusion of various mobility signals to sgNbPDS and sgZmHKT, respectively (Figures 1, 2). These results highlight the need to understand how RNA mobility signals, such as AtFT, function and how they can be used to facilitate viral delivery of sgRNAs to induce germline mutations. Furthermore, our work highlights the need to elucidate mechanisms by which viruses may naturally enter meristematic cells that give rise to progeny carrying mutations in target genes.

AtFT is a phloem mobile signal that promotes the transition from vegetative growth to flowering (Li et al., 2009; Lu et al., 2012) by moving from the leaves into the SAM (Foster et al., 2002; Kragler, 2010). Phloem targeting motifs are located at the 5′ end of the FT mRNA (Lu et al., 2012) as part of a photoperiod sensitive flowering signaling cascade (Jaeger and Wigge, 2007). The truncated 102 nucleotide fragment from the 5′ end of the FT mRNA was reported to direct localization of other coding and viral RNA sequences into the SAM (Li et al., 2011). FT orthologs have been identified in many plant species including *Solanum lycopersicum*, *O. sativa* and *Z. mays* suggesting that its function could be conserved (Lifschitz et al., 2006; Tamaki et al., 2007; Meng et al., 2011). Additionally, tRNA-like structures have been shown to be systemically transported throughout plants in grafting experiments that found tRNA movement from leaf to root and vice versa (Zhang et al., 2016). Strategically fusing mobility signal motifs to virus-produced sgRNAs is proposed to traffick these heterologous RNAs through the phloem and into the SAM, a highly protected region of pluripotent cells. The need for these mobility signals to permit virus-induced germline mutations would be most critical for plant viruses that are excluded from the SAM. In this study, some of the mobility signals we tested enhanced somatic editing in *Z. mays* when fused to sgRNAs delivered by FoMV, but they did not promote biallelic editing in progeny to a detectable frequency. It is unclear if mobility signals enabled FoMV or sgRNAs to access the SAM. Future *in situ*-based experimentation is needed to determine if mobility signals such as AtFT assist potexviruses such as FoMV to infect meristematic tissues.

In addition to plant factors, viral factors can also have a role in allowing a virus to access the meristem. For example, viral suppressors of RNA silencing (VSR) are capable of overcoming antiviral barriers in the SAM (Bradamante et al., 2021), and could be adopted from known seed transmissible viruses to existing systems to facilitate germline mutations. In BSMV and TRV, VSRs have been identified. The BSMV γb protein mediates vertical transmission, and the 16K protein from TRV is involved in meristem invasion, ultimately resulting in seed transmissibility (Bradamante et al., 2021). The SAM utilizes several defense mechanisms to preclude viral invasion including the expression of resistance genes, autophagy, and RNA interference (Bradamante et al., 2021). Another mechanism that was recently found to permit SAM entry, involved silencing of the WUSCHEL (WUS) transcription factor, which normally protects the SAM from virus infection by inhibiting viral protein synthesis, which results in broad-spectrum protection against viral infection (Wu et al., 2020). RNA viruses were able to gain access to the SAM in *A. thaliana* when WUS was suppressed (Wu et al., 2020). Utilization of heterologous VSRs, possibly in combination with WUS suppression, could enhance the ability of non-seed transmissible viruses to induce germline mutations, or improve current virus-induced germline mutation efficiency in systems for crop engineering. The prospect of obtaining germline mutations using diverse viral delivery systems appears to be more convoluted than simply fusing mobility signals to sgRNAs. We expect that host and viral cofactors cooperate with the mobility signals to play key roles in facilitating germline mutations.

FoMV, like most other potexviruses, is mechanically transmitted, but it is not generally considered to be seed transmissible. Vertical transmission of viruses occurs internally through maternal or paternal tissues, ultimately infecting the embryo (Sastry, 2013) as exemplified by BSMV, which is an internally seed transmitted virus (Carroll and Mayhew, 1976). Viral components associated with vertical transmission could facilitate heritability of gene edits, in line with the first two successful reports of viral-delivered sgRNAs producing germline mutations using the seed transmissible viruses, TRV (Ellison et al., 2020) and BSMV (Li et al., 2021; Chen et al., 2022). Previous data suggests that PVX can only be trafficked into the SAM when it carries full-length or truncated forms of AtFT (Li et al., 2011). Unexpectedly, we found that NbCas9 plants infected by PVX expressing the sgRNA targeting *NbPDS* alone produced progeny carrying biallelic edits in *NbPDS* (Figure 3; Supplementary Figure S4). This observation indicates that under our growth conditions PVX, or the subgenomic RNA carrying the sgRNA, possesses the ability to enter cells that give rise to the germline, possibly the SAM. In one study, the ectopic expression of the movement protein TGB1 of a closely related potexvirus, white clover mosaic virus (WClMV), allowed viral entry into the meristem (Foster et al., 2002). WClMV TGB1 acts as a VSR, and presumably compromises the plant’s ability to restrict viral entry into the SAM. The TGB1 of PVX has also been found to have VSR functionality (Bayne et al., 2005). However, in contrast to PVX, FoMV did not produce progeny with biallelic edits, suggesting that FoMV may not have the same capacity as other viruses of its genus. In compilation with other studies, our findings suggest that PVX, and perhaps other potexviruses, may possess alternative strategies to overcome meristem antiviral exclusion.

## Conclusion

In this study, we were interested in expanding the utility of the broad host range virus, FoMV, for germline mutations. However, it was unable to induce detectable biallelic progeny with or without mobility signals. This observation leads us to consider the possibility that RNA mobility signals, such as FT and tRNA, may not be sufficient to facilitate viral invasion of the cell types necessary to yield germline mutations, as postulated in previous studies. Our results, coupled with conflicting reports of the benefit of RNA mobility signals in BSMV-induced germline mutations (Li et al., 2021; Chen et al., 2022), demonstrate that there is still much to learn about the requirements for virus-induced germline mutations. Finding compatible virus-host combinations that ultimately gain meristem entry, and result in high levels of edited progeny is currently a challenging task. Therefore, more knowledge surrounding mechanisms of viral meristem exclusion and entry, and the interplay with VSRs is still needed to help researchers design effective delivery systems for virus-induced gene editing components in a broad range of plant species.

## Data Availability

The original contributions presented in the study are included in the article/Supplementary Material, further inquiries can be directed to the corresponding author.
