# Supplementary figures and images for: Impacts of RNA Mobility Signals on Virus Induced Somatic and Germline Gene Editing
Source: Front Genome Ed. 2022 Jun 9;4:925088. doi: 10.3389/fgeed.2022.925088 (PMC9219249; doi:10.3389/fgeed.2022.925088)

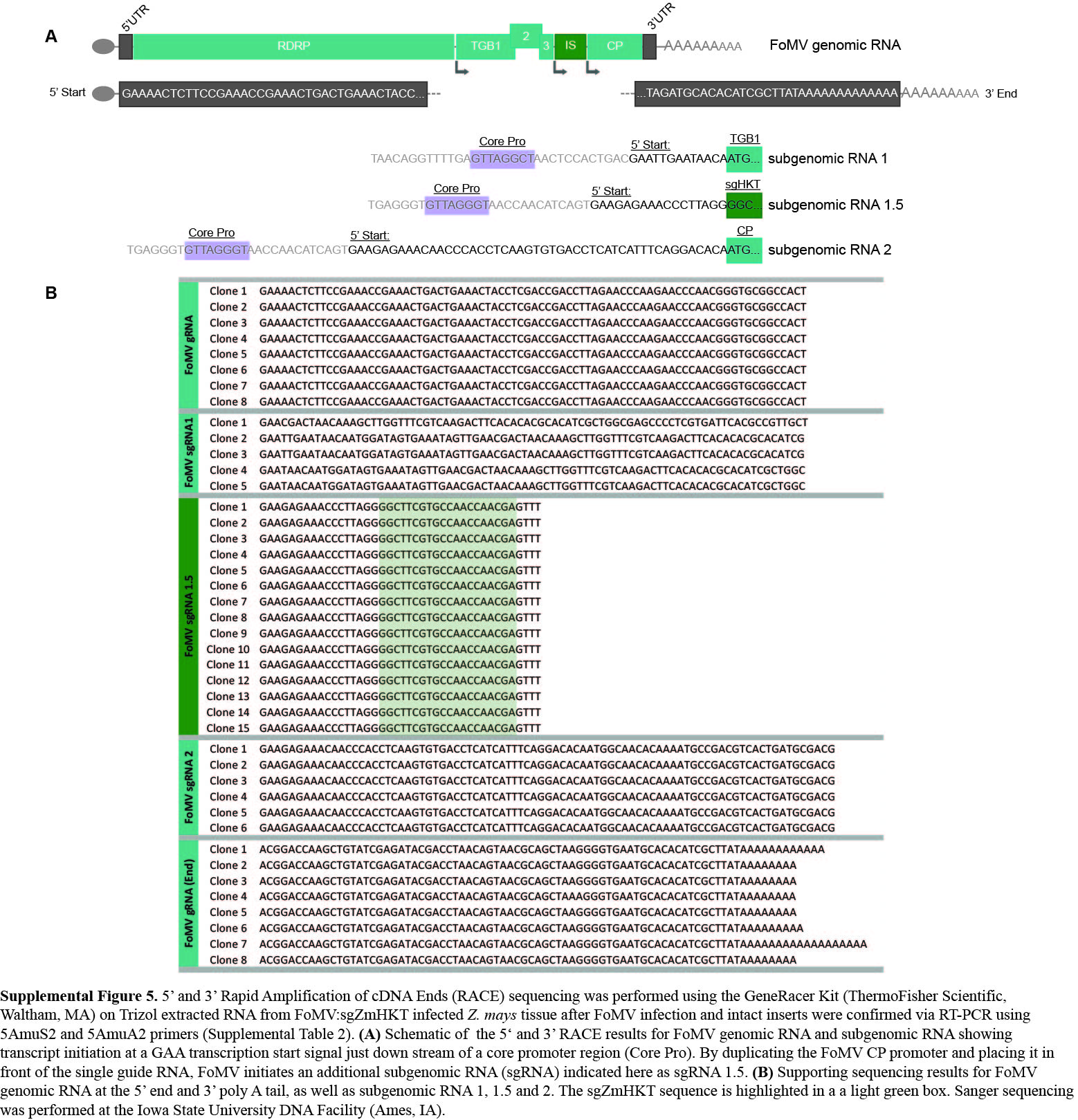

Supplement: Supplementary file 2 [file Image5.jpg]

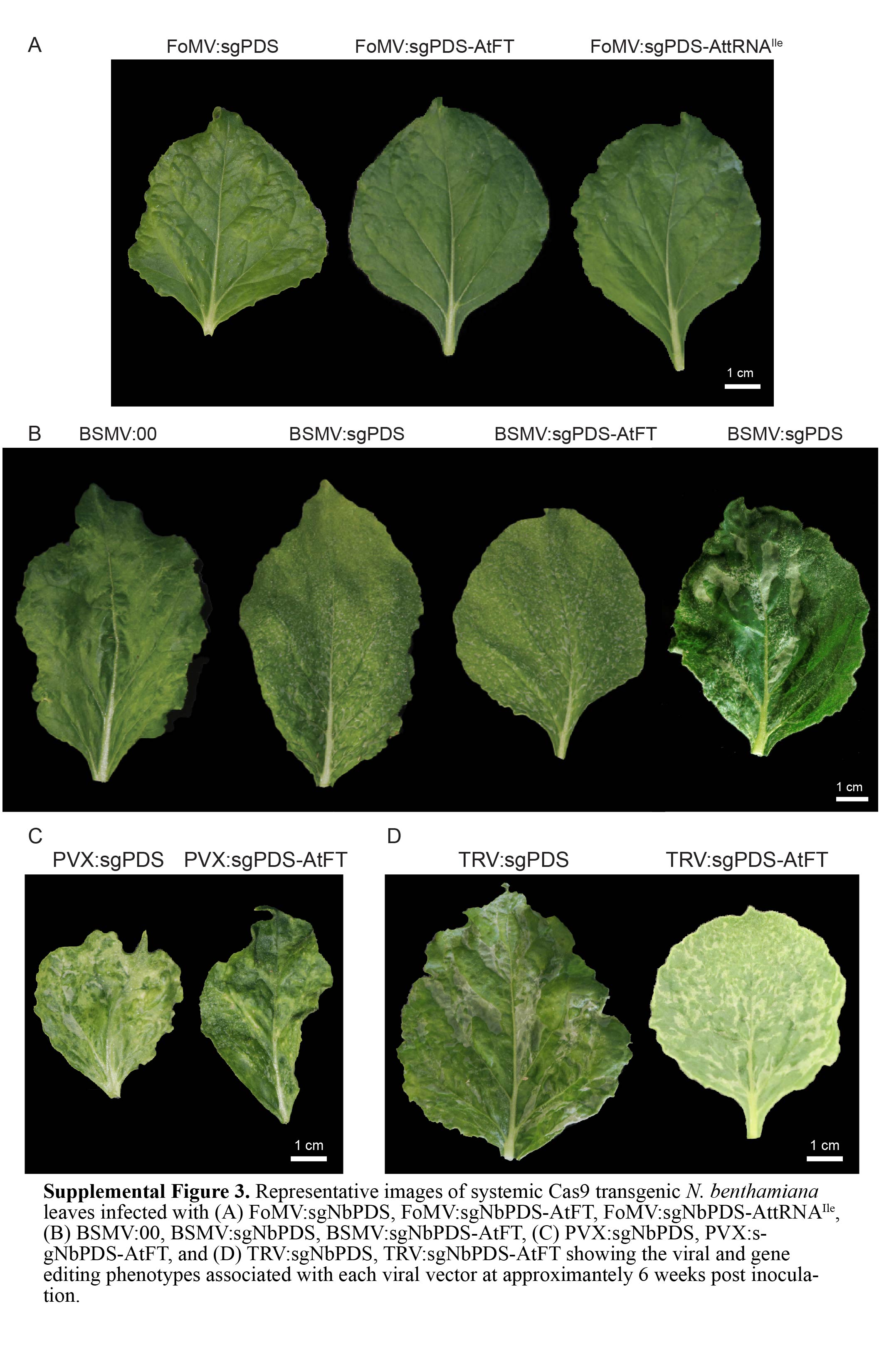

Supplement: Supplementary file 3 [file Image3.JPEG]

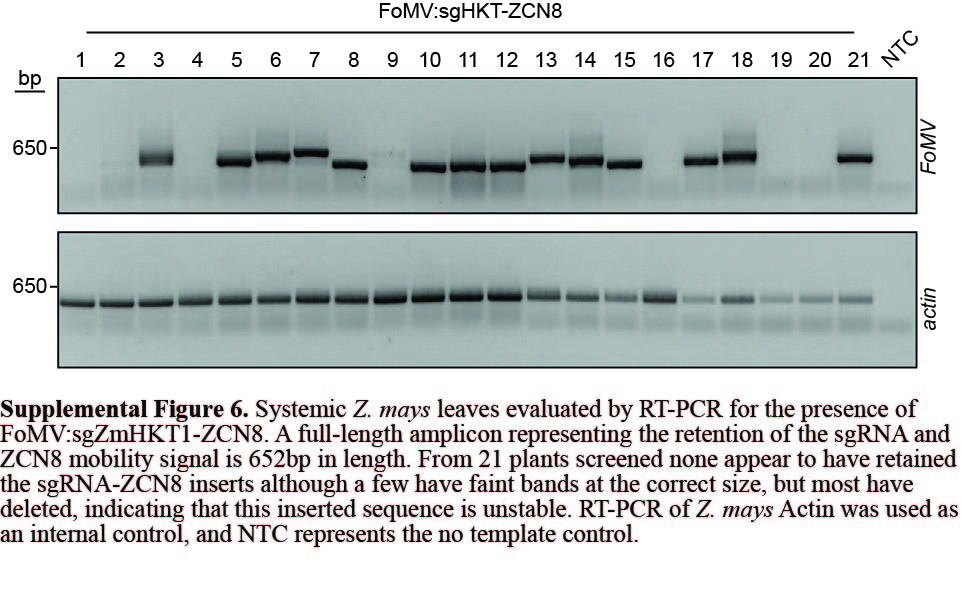

Supplement: Supplementary file 4 [file Image6.jpg]

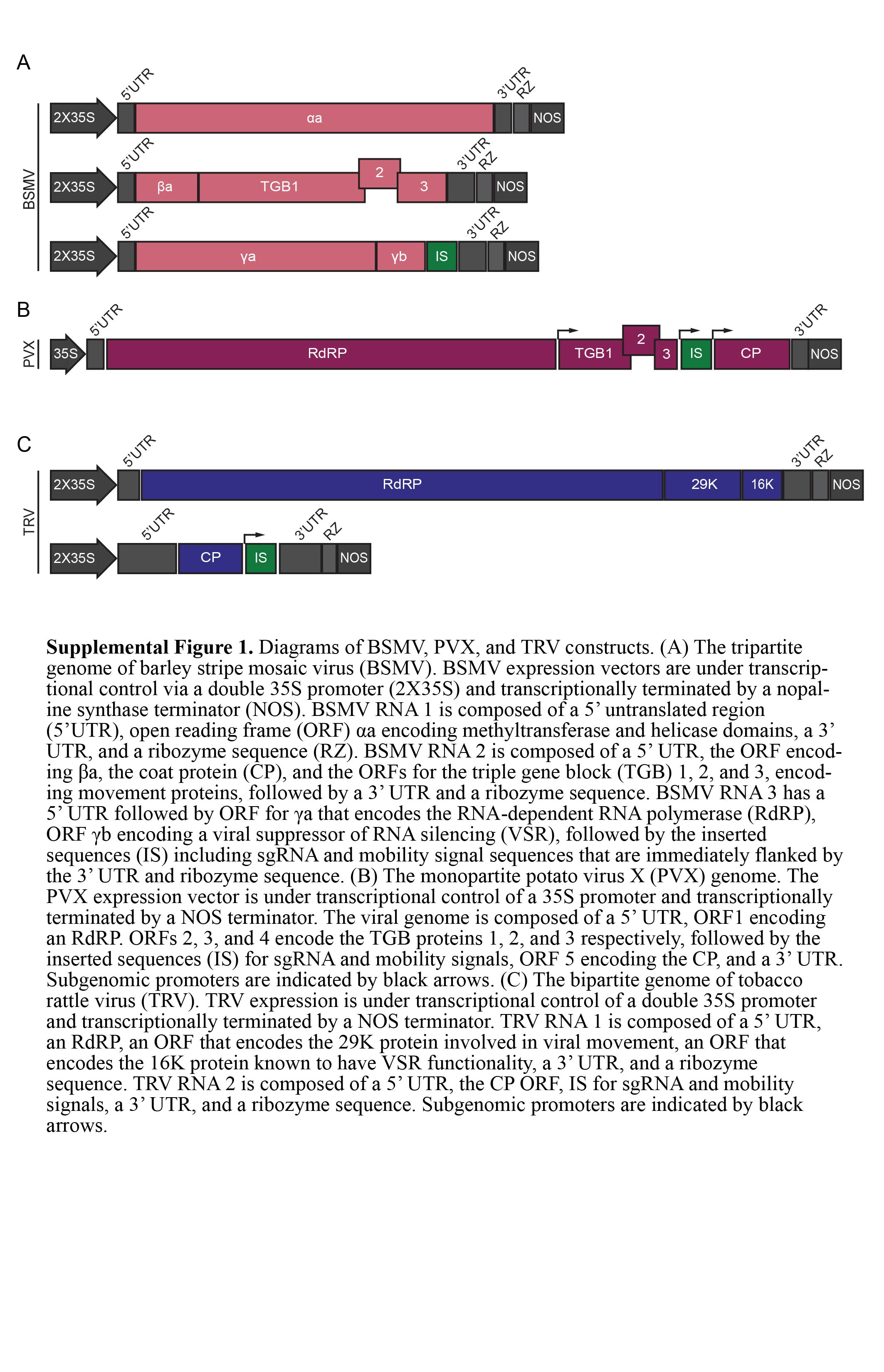

Supplement: Supplementary file 5 [file Image1.JPEG]

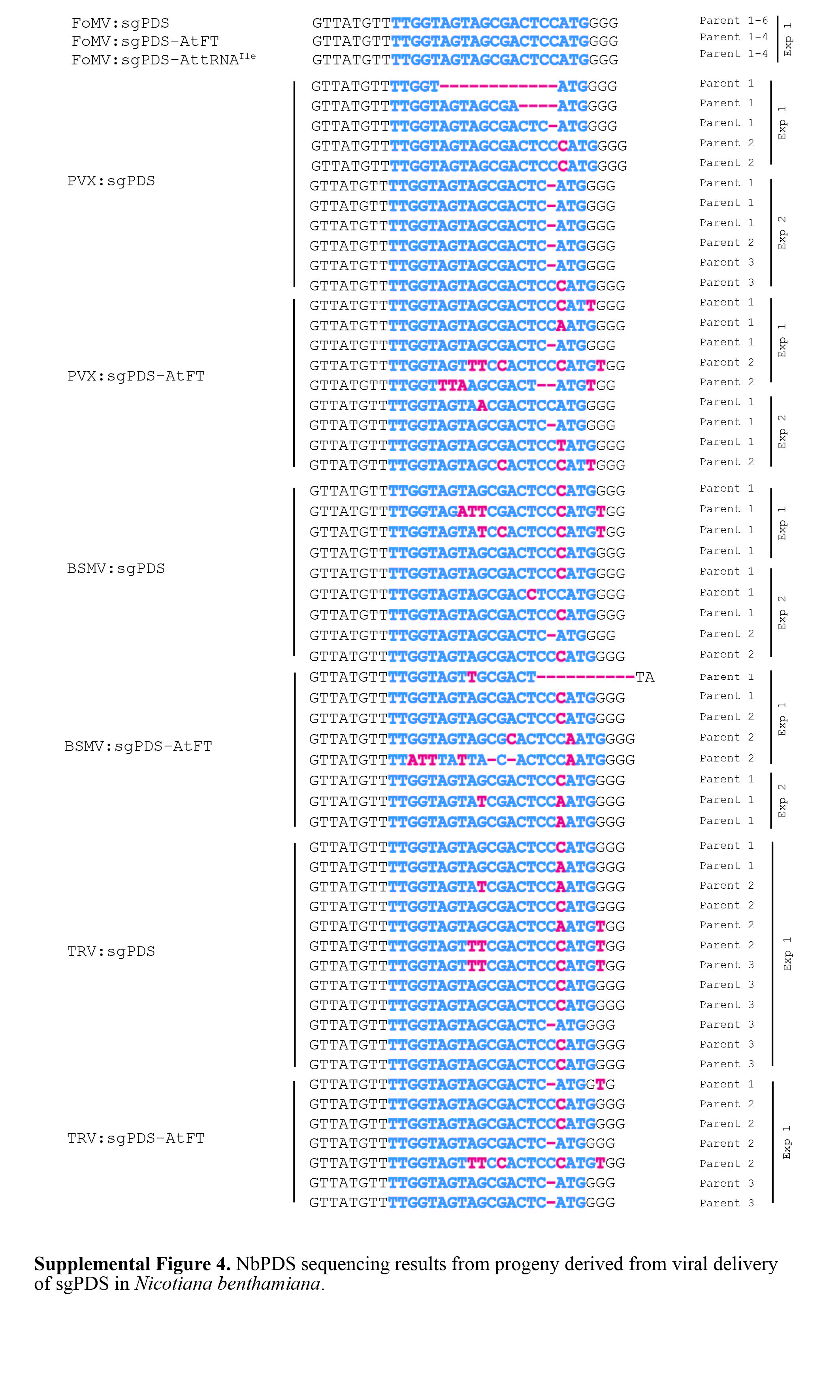

Supplement: Supplementary file 6 [file Image4.JPEG]

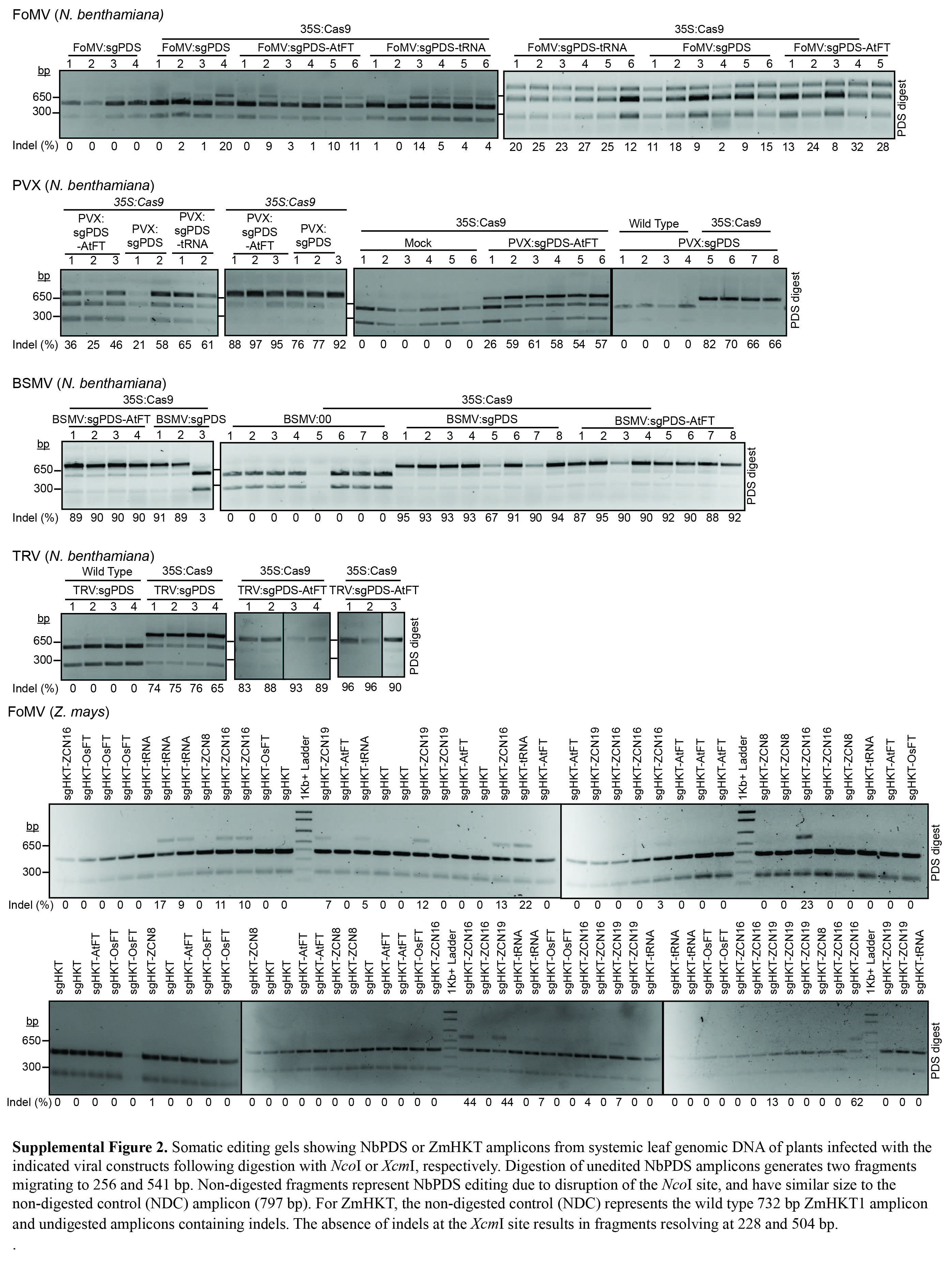

Supplement: Supplementary file 7 [file Image2.JPEG]
